# The characterization of *Moraxella catarrhalis* carried in the general population

**DOI:** 10.1099/mgen.0.000820

**Published:** 2022-05-26

**Authors:** Denise E. Morris, Karen L. Osman, David W. Cleary, Stuart C. Clarke

**Affiliations:** ^1^​ Faculty of Medicine and Institute for Life Sciences, University of Southampton, Southampton SO17 1BJ, UK; ^2^​ Global Health Research Institute, University of Southampton, Southampton SO17 1BJ, UK; ^3^​ NIHR Southampton Biomedical Research Centre, University Hospital Southampton Foundation NHS Trust, Southampton SO16 6YD, UK

**Keywords:** AMR, carriage, epidemiology, *Moraxella catarrhalis*, virulence

## Abstract

*
Moraxella catarrhalis* is a common cause of respiratory tract infection, particularly otitis media in children, whilst it is also associated with the onset of exacerbation in chronic obstructive pulmonary disease in adults. Despite the need for an efficacious vaccine against *M. catarrhalis *, no candidates have progressed to clinical trial. This study, therefore, aimed to characterize the diversity of *M. catarrhalis* isolated from the upper respiratory tract of healthy children and adults, to gain a better understanding of the epidemiology of *M. catarrhalis* and the distribution of genes associated with virulence factors, to aid vaccine efforts. Isolates were sequenced and the presence of target genes reported. Contrary to prevailing data, this study found that lipooligosaccharide (LOS) B serotypes are not exclusively associated with 16S type 1. In addition, a particularly low prevalence of LOS B and high prevalence of LOS C serotypes was observed. *M. catarrhalis* isolates showed low prevalence of antimicrobial resistance and a high gene prevalence for a number of the target genes investigated: *ompB2* (also known as *copB*), *ompCD*, *ompE*, *ompG1a*, *ompG1b*, *mid* (also known as *hag*), *mcaP*, *m35*, *tbpA*, *lbpA*, *tbpB*, *lbpB*, *msp22*, *msp75* and *msp78*, *afeA*, *pilA*, *pilQ*, *pilT*, mod, *oppA*, *sbp2*, *mcmA* and *mclS*.

## Data Summary

All genomes have been deposited in GenBank under BioProject ID PRJEB39742: accession numbers SAMEA7160394, SAMEA7160393, SAMEA7160392, SAMEA7160391, SAMEA7160390, SAMEA7160389, SAMEA7160388, SAMEA7160387, SAMEA7160386, SAMEA7160385, SAMEA7160384, SAMEA7160383, SAMEA7160382, SAMEA7160381, SAMEA7160380, SAMEA7160379, SAMEA7160378 and SAMEA7160377 (https://www.ncbi.nlm.nih.gov/biosample?Db=biosample&DbFrom=bioproject&Cmd=Link&LinkName=bioproject_biosample&LinkReadableName=BioSample&ordinalpos=1&IdsFromResult=655879).

**Impact Statement**

This paper gives new insight into the diversity and epidemiology of *Moraxella catarrhalis *, an increasingly important opportunistic human pathogen. These data help to clarify the distribution of 16S type, lipooligosaccharide (LOS) type and multilocus sequence type in community settings, and provide novel insight into the prevalence of antibiotic resistance and virulence factors in isolates circulating in the general healthy population. Contrary to prevailing data, this study found that LOS B serotypes are not exclusively associated with 16S type 1. In addition, a low prevalence of LOS B and a high prevalence of LOS C serotypes was observed. *M. catarrhalis* isolates showed a low prevalence of antimicrobial resistance and a high gene prevalence for a number of the target genes investigated. This is, to our knowledge, the first study to focus on carriage isolates, especially using strains isolates from people of all ages, and should now be followed by a similar analysis on a larger set of community isolates.

## Introduction

*Moraxella catarrhalis* is a Gram-negative, non-encapsulated diplococci and opportunistic human pathogen [1]. A common commensal of the upper respiratory tract [2], *M. catarrhalis* was once considered non-pathogenic. However, in the 1970s and 1980s, the pathogenic potential of *M. catarrhalis* was demonstrated through isolation from cases of disease [3–9]; it is now recognized as one of the most common causes of respiratory tract infection (RTI) [10, 11]. As the third largest bacterial cause of otitis media (OM) [12], *M. catarrhalis* causes 709 million cases of acute OM (AOM) globally each year; 51 % of which are in those ages four and under [13]. AOM is considered one of the most prevalent childhood conditions, with approximately 80 % of all children suffering at least one episode of AOM by 3 years of age [14, 15]. *M. catarrhalis* is also the second most common cause of exacerbation in chronic obstructive pulmonary disease (COPD) [15, 16], which is the third largest cause of global morbidity, responsible for 3 million deaths in 2016 [17].

*M. catarrhalis* has two distinct lineages that evolved independently, whilst divergent strains with lower homology have also been identified. Lineage one is complement resistant and adheres to epithelial cells; thus, it is known as the seroresistant subpopulation [18, 19]. Lineage two is less pathogenic, adheres less efficiently to the epithelium and is commonly complement sensitive; thus, it is known as the serosensitive subpopulation [18, 20, 21]. The seroresistant lineage comprises of 16S type 1 isolates, whereas 16S type 2 and 3 isolates fall into the serosensitive lineage [19, 21]. Whilst disease burden is greater from strains belonging to the seroresistant lineage, all 16S types can cause disease [19, 22]. Despite their separate evolution, distinct core genomes and differing genome size (~1.89 Mb for the seroresistant lineage, ~1.93 Mb for the serosensitive), both lineages show regular horizontal gene transfer [19]. *M. catarrhalis* is commonly typed according to the expression of highly conserved lipooligosaccharide (LOS) surface antigens, which forms the basis for the classification of *M. catarrhalis* into serotypes A, B or C [23, 24].

In recent years, the need for an efficacious vaccine against *M. catarrhalis* has been highlighted, yet no vaccine candidates have progressed to clinical trial [25, 26]. To help identify suitable vaccine targets, a better understanding of the epidemiology of *M. catarrhalis* and the distribution and diversity of virulence factors is required. Current data regarding the prevalence of *M. catarrhalis* and the distribution and expression of virulence genes across carriage and disease isolates remains inconclusive [18, 21, 22, 27]. For example, the gene for ubiquitous surface protein A1 (UspA1) has been reported present in 87–98 % of 16S type 1 isolates and 23–36 % 16S type 2 and 3 isolates [18, 21] in some research, whilst it is reported to be present in 100 % of 16S type 1 and >89 % of 16S type 2 and 3 isolates in other studies [22, 27]. However, the expression of *uspA1* appears similar in carriage and disease with expression of *uspA1* in 95 % of *M. catarrhalis* isolated in child carriage versus 97 % expression in *M. catarrhalis* isolated from child RTI [22].

Furthermore, whilst previous studies suggest 16S type 1 is most commonly associated with disease [18], it is unknown whether pathogenesis is implicitly associated with a particular type, what the roles of additional subpopulations or strains of *M. catarrhalis* are in disease epidemiology, or indeed what can be considered as the gene repertoire for virulence [28]. For example, most *M. catarrhalis* isolates, from 16S types 1, 2 and 3 (both seroresistant and serosensitive isolates), contain conserved genes for the majority of known virulence factors, suggesting perhaps all *M. catarrhalis* strains/16S types have equal pathogenic potential [19]. For instance, Uspa2 is vital for serum resistance, yet the gene encoding it is equally present in serum-resistant and serum-sensitive strains [21]. Similarly, the gene for *M. catarrhalis* immunoglobulin D binding outer membrane protein (MID) (also known as haemagglutinin/Hag) [29] is present in at least 90 % of child RTI isolates, 91 % of adult RTI isolates and 80 % of child carriage isolates, indicating no clear link between gene presence and carriage or disease [22, 27, 29]. This highlights the importance of looking at the genotypes and phenotypes of both disease and carriage isolates. As virulence is based on multiple factors, the balance between the harmless carriage of *M. catarrhalis* and the development of disease may be determined by the combination of the virulence genes present, differences in expression of these genes and environmental factors [19]. Understanding the prevalence and distribution of numerous virulence factors and their importance in carriage and disease and, thus, their potential use in vaccine development is vital.

The aim of this study was to characterize a collection of *M. catarrhalis* isolated from people of all ages, to give new insight into the diversity and epidemiology of this important human pathogen. As the first study, to our knowledge, to focus on carriage isolates, especially using strains isolated from people of all ages, it provides important data to improve our knowledge of the diversity of *M. catarrhalis* and to update the literature.

## Methods

### Sample collection and bacterial identification

A large population-based cross-sectional carriage study, the ‘Analysis of the microbial community of the upper respiratory tract to support the development of effective vaccine policy’ study (Bupa SMART study; REC reference 11/SC/0518), was undertaken as described previously [30, 31]. Briefly, swabbing was undertaken over two time-points: May to August 2012 and February to April 2013. Study participants were identified from general practice lists in the Wessex Primary Care Research Network and were randomized into one of two study arms. For one arm, each participant took a self-taken nasal swab and for the other arm a nasopharyngeal (NP) swab was taken by a trained healthcare professional [30, 31]. Prior to culture, each swab was immersed and vortexed in skim milk, tryptone, glucose and glycerine (STGG) storage media. For each, 10 µl was plated onto Columbia blood agar with horse blood (Oxoid) and Columbia blood agar with chocolated horse blood (Oxoid). Plates were incubated for 24–48 h at 37 °C in 5 % CO_2_. *M. catarrhalis* were initially identified by standard morphology: non-haemolytic colonies that appear grey or white on blood agar or pinkish brown on chocolated agar, opaque, flat, smooth, dry, stay as complete colonies when pushed across agar and are 1–3 mm in diameter after 24 h of incubation [32]. Isolates were then verified as *M. catarrhalis* by being confirmed as Gram-negative, and oxidase, tributyrin and DNase positive. Tests were done using oxidase strips (Oxoid), tributyrin strips (Sigma- Aldrich) and DNase/methyl green plates (VWR; EOLAPP0560) as per manufacturers' instructions. Pure growth was frozen at −80 °C in STGG for future analysis.

### Isolates

A subset (*n*=24) of *M. catarrhalis* were selected for whole-genome analysis. Equal numbers (*n*=6) of *M. catarrhalis* were drawn from participants in the following age ranges; 0–4 years, 5–16 years, 17–59 years and 60+ years. *M. catarrhalis* isolated from NP swabs were preferentially chosen, as the nasopharynx is the recommended sampling site for these bacteria [33]. Where this was not possible, *M. catarrhalis* isolated from nasal swabs were used.

### Whole-genome sequencing

DNA extraction was performed for each isolate using an overnight culture of a single colony pick, grown on Columbia blood agar with horse blood (Oxoid). Here, the Qiagen mini prep kit (Qiagen) was used in accordance with the manufacturer's instructions. DNA quantification was done using a Qubit fluorometer (Invitrogen). DNA extracts were then diluted to 0.2 ng µl^−1^ in distilled water. Library preparation was done using a Nextera XT kit (Illumina). Sequencing was done in house using an Illumina MiSeq to generate 2×250 bp V2 paired-end read data.

### Bioinformatics

FastQC v. 0.11.5 (http://www.bioinformatics.babraham.ac.uk/projects/fastqc/) was used to assess read metrics. Reads were trimmed to remove adapters using Trimmomatic v. 0.32 [34]. Multilocus sequence type (MLST) was obtained by submitting the Fastq files to EnteroBase (https://enterobase.warwick.ac.uk/species/index/mcatarrhalis); accessed February 2017. Genome assembly was undertaken using SPAdes v. 3.1 [35] and the quality of assembly was checked using Quast v. 4.2 [36]. LOS serotyping was done with *in silico* PCR using Ipcress (with a primer mismatch tolerance of three) on assembled genomes, using published primer sequences [37]. Expected product lengths for serotypes A, B and C were 1.9, 3.3 and 4.3 kbp, respectively. 16S typing, a common method for the classification of *M. catarrhalis* based on the homology of 16S rRNA sequence, was similarly undertaken using Ipcress with published primers [21]. Resulting sequences were mapped against the 16S sequences of known types [21] using srst2 v. 0.1.3 [38]. ParSNP v. 1.2 [39] was used to align and construct a core-genome phylogeny, using BBH18 as a reference genome. The resultant phylogeny was visualized using iTOL version 4.2.3 [40].

The *M. catarrhalis* pangenome was defined and analysed using Anvi’o v7 with reference strains NCTC 11020 and BBH18 included for comparison [41]. Identification of ORFs and annotation with Clusters of Orthologous Genes (COGs) function was done within Anvi’o. Amino acid sequence comparisons were done using blastp using the --use-ncbi-blast flag with additional initial parameters including a minbit score of 0.5 and an Markov Clustering (MCL) inflation of 10 for clustering of genes into gene clusters. Functional enrichment analysis was done using the ‘anvi-get-enriched-functions-per-pan-group’ using the COGs as the function annotation source. This contrasts the prevalence of gene clusters and associated functional annotation, rather than genes, between user-defined groups of isolates using a generalized linear model with the logit linkage. This outputs both an enrichment score and *P* value for each function. Benjamini–Hochberg false discovery rate (FDR) corrected *P* values (<0.05) were used to determine enriched functions between phylogenetic clades. Finally, average nucleotide identity (ANI) was computed using ‘anvi-compute-genome-similarity.

### Antibiotic resistance

srst2 v. 0.1.3 [38] using the ARGannot.r1.fasta database for acquired resistance genes and ResFinder v. 2.1 (https://cge.cbs.dtu.dk/services/ResFinder/; accessed May 2021 with an 80 % ID threshold) [42] were used to detect the presence of antibiotic-resistance genes. Additionally, reads were mapped, using srst2 v. 0.1.3 [38], to the *bla BRO-1* (GenBank accession no. Z54180.1) and *bla BRO-2* (GenBank accession no. Z54181.1) gene sequences, which can confer resistance to β-lactam antibiotics as they code for the production of β-lactamase. Consensus sequences from srst2 were aligned in Clustal Omega [43] for manual confirmation of gene presence, the sequences obtained were verified as *bla BRO-1* or *bla BRO-2* using blast (https://blast.ncbi.nlm.nih.gov/Blast.cgi).

Antibiotic resistance was also tested phenotypically. Here, 10 µl of each isolate (a suspension of cells in liquid STGG) was plated onto Columbia blood agar with horse blood (Oxoid). Plates were incubated for 24 h at 37 °C in 5 % CO_2_. Pure colonies were added to 1 ml saline to get an inoculum of 0.5 McFarland. A sterile swab was used to spread this inoculum over Mueller Hinton F plates [Mueller-Hinton agar, 5 % defibrinated horse blood and 20 mg β-NAD l^−1^ (Oxoid)]. Antibiotic discs, four per plate, were added and plates were incubated at 35 °C in 5 % CO_2_ for 18 h (±2 h). Each *M. catarrhalis* isolate was tested with amoxicillin/clavulanic acid, 2/1 μg; cefotaxime, 5 μg; ceftriaxone, 30 μg; erythromycin, 15 μg; tetracycline, 30 μg; chloramphenicol, 30 μg; ciprofloxacin, 5 μg; and meropenem, 10 μg (all from Oxoid). EUCAST (European Committee on Antimicrobial Susceptibility Testing) breakpoints were used to assess susceptibility and resistance. β-Lactamase production was confirmed using β-lactamase identification sticks (Oxoid), as per the manufacturer's instructions [44].

### Virulence factors

Isolates were tested for the presence of genes for UspA1, UspA2 and UspA2H, outer membrane proteins (OMP) B2 (also known as C̱atarrhalis o̱uter membrane p̱rotein Ḇ/CopB), CD, E, G1a and G1b, MID/Hag, *M. catarrhalis* adherence protein (McaP), outer membrane porin M35, *Moraxella* haemagglutinin-like proteins (Mha) B1, B2 and C; transferrin binding proteins (Tbp) A and B, lactoferrin binding proteins (Lbp) A and B, *Moraxella* surface proteins (Msp) 22, 75 and 78, chelated iron ABC transporter substrate binding protein AfeA, *M. catarrhalis* metallopeptidase-like adhesin (McmA), *M. catarrhalis* cardiolipin synthase (MclS), *M. catarrhalis* type IV pilin (PilA), *M. catarrhalis* type IV pilus biogenesis secretin (PilQ), *M. catarrhalis* type IV pilus retraction ATPase (PilT), *M. catarrhalis* type III restriction-modification system methyltransferase (ModM), oligopeptide permease protein A (OppA) and substrate binding protein 2 (SBP2). This was done by read mapping of raw sequence reads (.fastq.gz) using BBMap v36.59 [45] and BWA v0.7.17 [46] against the following NCBI, EMBL or ENA references: AF113610.1, EU430059.1, U61725.1, AF113610.1 and AF352398.1 (*uspA1*), AY730666.1, AF352399.1, AF410950.1, AF352399.1, AF181073.1 and AF113609.1 (*uspA2*), AF181075.1, DQ811779.1, AF410951.1, AF181074.1 and AF181075.1 (*uspA2H*), AY726667.1, AY726666.1 and AY726664.1 (*copB*), AY493741.1 (*ompCD*), L31788.1 and CP002005.1 (*ompE*), AY275816.1 and AY275809.1 (*ompG1a*), AY462077.1, AY462072.1 and AY462066.1 (*ompG1b*), AY862881.1, AY077638.2 and AY077637.1 (*mid/hag*), EF075933.1, EF075940.1 and EF075937.1 (*mcaP*), AY905613.1 (*m35*), EF362385.1 and EF362386.1 (*mhaB1*), EF362388.1 and EF362389.1 (*mhaB2*), EF362396.1 and EF362391.1 (*mhaC*), AF039315.1 (*tbpAB*), AAC34275.1 (*tbpA*), AF039314.1 and AF039311.1 (*tbpB*), AF043131.1 (*lbpAB*), AAC31366.1 (*lbpA*), AF043133.1 (*lbpB*), ACA52193.1 (*msp22*), EU339314.1 (*msp75*), EU339313.1 (*msp78*), EMBL ADG62123.1 (a*feA*), EF017300.1 (*mcmA*), KC692996.1 (*mclS*), AY647185.1 and HQ442298.1 and ADG60664.1 (*pilA*), AY647186.1 (*pilQ*), AY647187.1 (*pilT*), AY049056.1 and CP018059.1 (*modM*), ADG61563.1 (*oppA*) and STY81838.1 (*sbp2*). SAMtools v0.1.19 [47] was used to view, sort and index .bam files. Coverage statistics were generated with pileup. Cut-offs for sequence coverage were specific for each gene, based on the level of variation expected for each based on prior publication as detailed in Table 1. Therefore, genes known to be less conserved were permitted a lower sequence coverage as an indication of gene presence.

**Table 1.** Gene identity and cut-off points for read mapping analysis

Gene	Identity (%)	Reference	Cut-off used (%)
*hag*	56.6–85	[24, 28]	55
*uspA1*	Modular	[28]	–
*uspA2*	Modular	[28]	–
*uspa2H*	Modular	[28]	–
*modM*	70	[28]	70
*mhaB1*	68.8	[28]	68
*mhaB2*	98	[28]	90
*mhaC*	Not published	–	70
*tbpA*	98	[28]	90
*tbpB*	51	[28]	50
*lbpA*	99	[28]	90
*lbpB*	77	[28]	75
*pilA*	>78	[28, 82]	70
*pilQ*	Not published	–	70
*pilT*	Not published	–	70
*ompG1a*	90	[28]	90
*ompG1b*	92	[28]	90
*mcmA*	Not published	[28]	70
*sbp2*	99.8	[28]	90
*copB*	76.1	[28]	70
*ompCD*	97.1	[28]	95
*ompE*	96.6–100	[28]	97
*mcaP*	98–100	[28]	98
*m35*	92.8–99.4	[28]	90
*msp22*	99	[28]	90
*msp75*	97	[28]	90
*msp78*	99	[28]	90
*afeA*	87–100	[28]	85
*mclS*	99	[83]	90
*oppA*	98.7	[28]	90

Whilst the current literature suggests *mhaB2*, *tbpA*, *lpbA*, *SBP2*, *ompE*, *ompCD*, *mcaP*, *msp78*, *mclS* and *oppA* all have conservation identities of >95 %, a 90 % coverage cut-off was used to ensure genes showing slight variation were not excluded. A 90 % coverage means the reads overlap 90 % the length of the gene supporting the presence of the gene.

### Reference strains

Reference strains NCTC 11020 and BBH18 were included for comparison in figures and as controls to assess the accuracy of bioinformatic analysis. However, the data presented within the text of the results are solely for the *M. catarrhalis* isolated as part of the SMART study; thus, data are representative of carriage isolates only. Whilst BB818 was included as a reference as a known serum-resistant strain, no data on serum sensitivity was available for NCTC 11020. NCTC 11020 has previously been sequenced and this data published, so was included as an additional reference to be used as a quality control for in-house sequencing.

## Results and Discussion

### Carriage of *M. catarrhalis*

From the 314 NP swabs obtained during the Bupa SMART study, 14 *M *. *catarrhalis* were isolated representing an overall carriage prevalence of 4.5 %. Of the 314 participants who had NP swabs taken, 56 were 0–4 years, 24 were aged 5–16, 59 were aged 17–59 and 175 were aged 60 or over. The NP carriage prevalence of *M. catarrhalis* for each age group was 10.7 % (6/56) for those aged 0–4, 4.2 % (1/24) for those aged 5–16, 3.4 % (2/59) for those aged 17–59 and 2.9 % (5/175) for those aged 60 years and over. Of the 2103 nasal swabs received, 96 *M *. *catarrhalis* were isolated: an overall carriage prevalence of 4.6 %. Of the 2103 participants who took nasal swabs, 497 were 0–4 years, 248 were aged 5–16, 614 were aged 17–59 and 708 were aged 60 or over. No age was provided by 36 participants. The nasal carriage prevalence of *M. catarrhalis* for each age group was 10.1 % (50/497) for those aged 0–4, 6.9 % (17/248) for those aged 5–16, 1.5 % (9/614) for those aged 17–59 and 2.4 % (17/708) for those aged 60 years and over. All NP swabs were taken between May and August 2012 (the first time-point). Nasal swabs were taken over both time-points; 1260 nasal swabs were taken between May and August 2012 and 843 were taken between February and April 2013 [30, 31].

Of the 24 pre-selected *M. catarrhalis* isolates, 18 were successfully sequenced; the number of contigs ranged from 27 to 141 with a mean of 58.9, whilst the N50 ranged from 31 207 to 214 693 with a mean of 108 622.8. Metadata for these isolates, including information about the participants from whom they were obtained, can be seen in Table 2.

**Table 2.** Isolate metadata

Participant data					* Moraxella catarrhalis *	
Isolate no.	Swab type	Age (years)	Antibiotic use*	Vaccination up to date	Date of culture (dd.mm.yy)	Co-carriage†
57	NP	2	No	Yes	07.06.12	None
626	NP	1	No	Yes	29.06.12	* S. pneumoniae *
628	NP	3	No	Yes	29.06.12	None
1077	NP	4	No	Yes	16.07.12	None
1080	NP	2	Yes (flucloxacillin)	Yes	16.07.12	None
1227	NP	1	No	Yes	24.07.12	* S. pneumoniae *
1343	Nasal	6	No	Yes	30.07.12	* H. influenzae* and *S. aureus *
1592	Nasal	5	No	Yes	19.02.13	* S. pneumoniae *
1648	Nasal	6	No	Yes	20.02.13	None
1833	Nasal	7	No	Yes	27.02.13	None
18	NP	26	No	Yes	02.07.12	None
608	Nasal	34	No	Yes	29.06.12	None
19	Nasal	41	No	No	05.03.13	None
20	Nasal	43	No	Yes	16.03.13	None
687	NP	85	No	Yes	03.07.12	None
10 309	NP	75	No	No	27.07.12	None
1470	NP	81	No	Yes	07.08.12	None
37	Nasal	81	No	Yes	24.05.12	None

*Indicates use in the month prior to swabbing.

†*H. influenzae*, *Haemophilus influenzae*; *S. aureus*, *Staphylococcus aureus*; *S. pneumoniae*, *Streptococcus pneumoniae*.

**Fig. 1.**
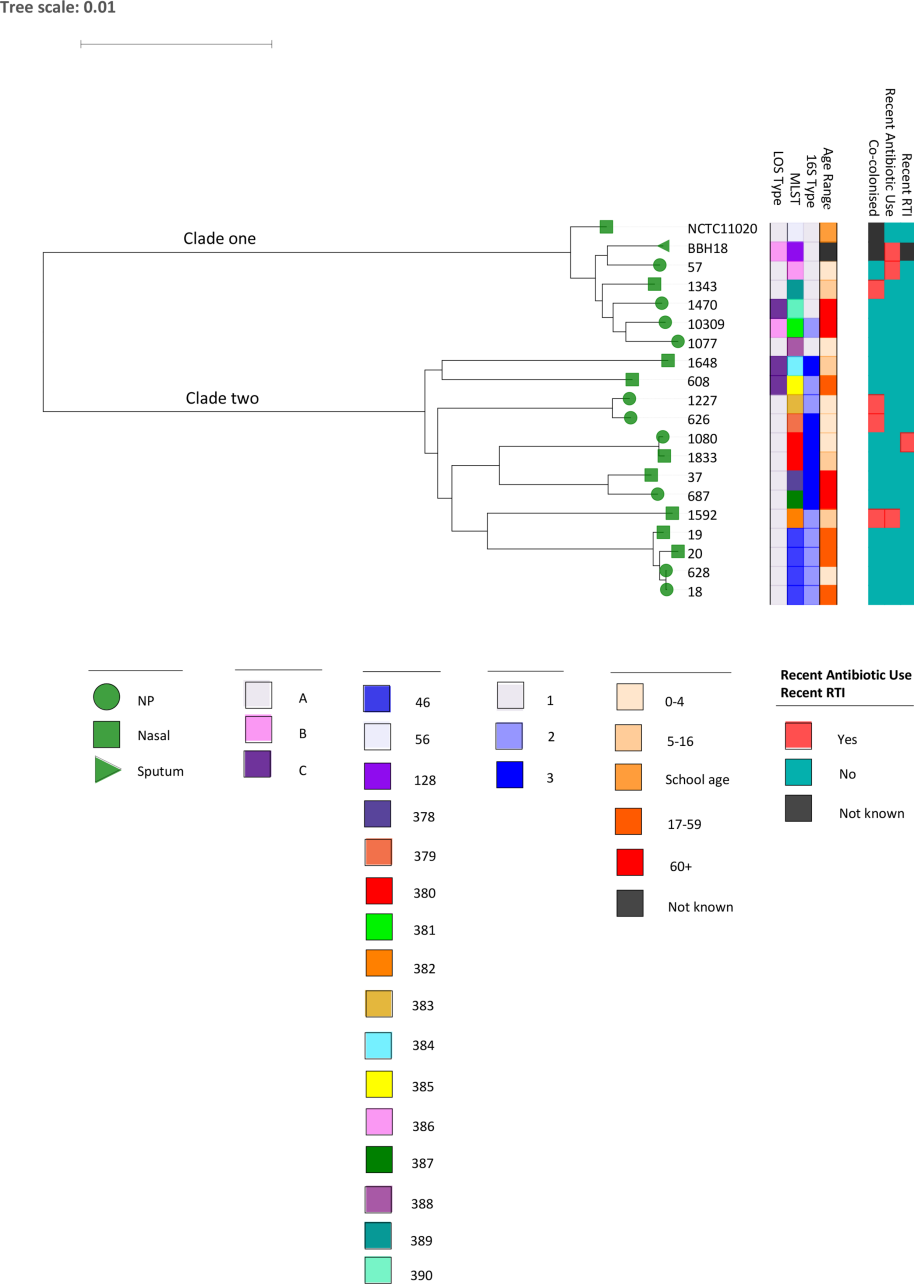
Core-genome phylogeny.

All 18 isolates were assigned a sequence type (ST) (Table 3); four isolates were ST46, all of which were LOS type A and 16S type 2. Two isolates were identified as ST380, both of which were LOS type A and 16S type 3. The remaining isolates were singleton STs. No clear correlation between ST and metadata was observed. Similarly, no clear correlation can be seen between LOS or 16S type and any of the metadata, as shown by the core-genome phylogeny in Fig. 1. As the subset of *M. catarrhalis* was chosen to ensure we had isolates obtained from participants in the following age ranges, 0–4 years, 5–16 years, 17–59 years and 60+ years, no associations can be made between carriage and prior antibiotic use, respiratory infection and vaccine status. All *M. catarrhalis* isolates from the study and all related metadata would be needed for such associations to be made.

**Table 3.** Virulence and typing results

Isolate	BBH18	NCTC 11020		57	1343	1470	10 309	1077	1648	608	1227	626	1080	1833	37	687	1592	19	20	628	18
*copB*																					
*ompCD*																					
*ompE*																					
*ompG1a*																					
*ompG1b*																					
*mid/hag*																					
*mcaP*																					
*m35*																					
*mhaB1*																					
*mhaB2*																					
*mhaC*																					
*tbpA*																					
*tbpB*																					
*lbpA*																					
*lbpB*																					
*msp22*																					
*msp75*																					
*msp78*																					
*afeA*																					
*mcmA*																					
*mclS*																					
*pilA*																					
*pilQ*																					
*pilT*																					
*modM*																					
*oppA*																					
*sbp2*																					
LOS type	B	A		A	A	C	B	A	C	C	A	A	A	A	A	A	A	A	A	A	A
MLST	128	56		386	389	390	381	388	384	385	383	379	380	380	378	387	382	46	46	46	46
16S type	1	1		1	1	1	2	1	3	2	2	3	3	3	3	3	2	2	2	2	2
β-Lactamase (*bla BRO*)	2			1	1	2	1	1	2	2	1	1	1	1	2	1	2	1	1	1	1
Age (years)		School age		2	6	81	75	4	6	34	1	1	2	7	81	85	5	41	43	3	26
Clade	1	1		1	1	1	1	1	2	2	2	2	2	2	2	2	2	2	2	2	2
							
Gene presence			LOS type		MLST	16S type		β-Lactamase gene			Age range (years)		Core-genome phylogeny clade								
Present			A		46	1		*bla BRO*-1			0–4		1								
Absent			B		56	2		*bla BRO*-2			5–16		2								
			C		128	3					17–59										
					378						60+										
					379																
					380																
					381																
					382																
					383																
					384																
					385																
					386																
					387																
					388																
					389																
					390																

### Distribution of LOS types

*In silico* analysis of the glycosyltransferase (*lgt*) genes showed that 77.8 % (14/18) of isolates were LOS type A, 5.6 % (1/18) were LOS type B and 16.7 % (3/18) were LOS type C. Overall, 90 % (9/10) of *M. catarrhalis* isolated from children were LOS A, with the remaining 10 % (1/10) being LOS C, whilst 62.5 % (5/8) of *M. catarrhalis* isolated from adults were LOS A, 12.5 % (1/8) were LOS B and 25 % (2/8) were LOS C. When distribution was considered by age range, all *M. catarrhalis* isolated from 0 to 4 year olds (6/6) were identified as LOS type A. Of the isolates from the 5–16 age group, 75 % (3/4) were LOS type A, with the remainder being LOS type C. This matches the data from 17 to 59 year olds for whom LOS type A represented 75 % (3/4) of the isolates with the remaining 25 % (1/4) being LOS type C. Of the four isolates from the 60+ age group, 50 % (2/4) were LOS type A, with 25 % (1/4) being type B and 25 % (1/4) type C.

Prior publications suggest that the majority of clinical isolates express LOS type A (61–70 %) while few express LOS type C (2–7 %) [22, 37, 48, 49]. The data here agree with regards to the majority prevalence of LOS type A, regardless of the age of the carrier. However, in contrast, our data show a much lower prevalence of LOS type B (5.6 % here versus 19–30 %) and a much higher prevalence of LOS type C (16.7 % here versus 2–7 %) [22, 37, 48, 49]. There are numerous reasons why the results in this dataset may differ to that previously published, firstly the low number of isolates in our dataset. Alternatively, isolates in numerous prior publications are now at least 16 years old [22, 37, 48]; therefore, previously published LOS prevalences may not be reflective of current epidemiology. Conversely, dissimilarities could be due to geographical differences in the distribution on LOS types, or differences in carriage versus clinical isolates.

Research using clinical isolates from the USA, showed higher proportions of LOS type A in *M. catarrhalis* isolates from adults versus isolates from children (81 % in adults versus 64 % in children) and a lower proportion of LOS type B in adults versus children (15 % in adults versus 34 % in children) [37]. However, our carriage data show an opposite trend, which is comparable to clinical data obtained globally, which observed that 81 % of *M. catarrhalis* from children were LOS type A whilst the percentage was lower (63%) in adults. This was reversed for LOS B where 13 % of isolates from children were LOS type B whilst the percentage was higher (28%) for isolates from adults, again fitting the trend (but not the values) seen in our data [22]. Consequently, there may be little difference in distribution of LOS in carriage and disease, as suggested by Mitov *et al*. [49]. This highlights the potential use of carriage data to provide insight into disease, certainly for LOS distributions. Furthermore, the majority of clinical isolates may be LOS type A, not because strains of this type are more pathogenic, but simply because they are more prevalent.

### Distribution of 16S types

In total, 22.2 % (4/18) of isolates were 16S type 1, 44.4 % (8/18) were 16S type 2 and 33.3 % (6/18) were 16S type 3. It has previously been reported that 92 % of clinical isolates are 16S type 1, 4 % type 2 and 4% type 3 [22]. The proportion of 16S type 1 seen here is substantially lower (22.2%) with a higher prevalence of type 2 and 3 isolates. These differences could be a result of our data being based on carriage isolates.

Overall, 30 % (3/10) of isolates from children were 16S type 1, 30 % (3/10) were 16S type 2 and 40 % (4/10) were 16S type 3, whilst 12.5 % (1/8) of isolates from adults were 16S type 1, 62.5 % (5/8) were 16S type 2 and 25 % (2/8) were 16S type 3. When distribution of 16S type is considered by set age range, an equal proportion of 16S types was found in young children (0–4 years old); 33.3 % (2/6) for each. In comparison, 25 % (1/4) of the *M. catarrhalis* from older children aged 5–16 were identified as 16S type 1, 25 % (1/4) as type 2 and 50 % (2/4) as type 3. All isolates (*n*=4) from adults aged 17–59 were 16S type 2. For the 60+ age range, 25 % (1/4) of isolates were identified as 16S type 1, 25 % (1/4) as type 2 and 50 % (2/4) as type 3.

### LOS versus 16S type

**Fig. 2.**
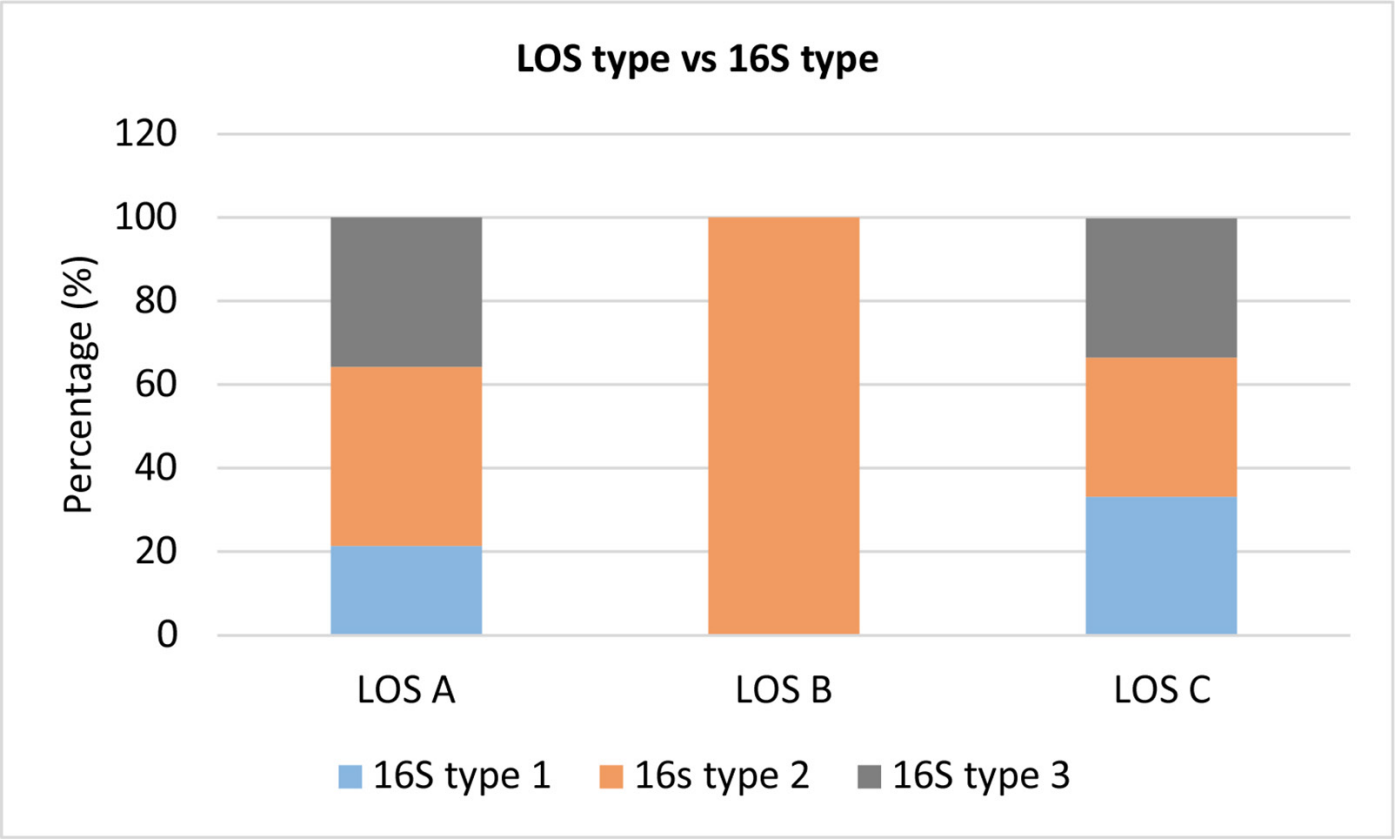
A graph illustrating the distribution of 16S types found for each LOS type.

From the 14 isolates identified as LOS type A, 21.4 % (3/14) were found to be 16S type 1, 42.9 % (6/14) were identified as 16S type 2 and 35.7 % (5/14) 16S type 3. The LOS type B isolate was found to be 16S type 2. From the 3 LOS type C isolates, 33.3 % (1/3) were identified as 16S type 1, 33.3 % (1/3) were identified as 16S type 2 and 33.3 % (1/3) identified as 16S type 3. This is visualized in Fig. 2.

Previous research found 91% of LOS type A *M. catarrhalis* were 16S type 1, 4 % were 16S type 2 and 5 % were 16S type 3 [22]. Here, however, a much lower proportion (21.4%) of the LOS type A isolates were also identified as 16S type 1 (3/14), with 42.9 % (6/14) being 16S type 2 and 35.7 % (5/14) identified as 16S type 3.

It was believed that LOS B was exclusively associated with 16S type 1, as previously 100 % of isolates were identified as such [22]. However, the LOS type B isolate from this study was identified as 16S type 2. This study has, therefore, importantly highlighted that LOS B serotypes may not exclusively be associated with 16S type 1, although further phenotypic analysis is required for confirmation of LOS expression.

Previous data show *M. catarrhalis* LOS type C isolates to have an even split, with one half reported as 16S type 1 and the other 16S type 2 [22]. Our data, however, suggest an even split across all 16S types with 33.3 % (1/3) being attributed to all three types.

### Pangenome

**Fig. 3.**
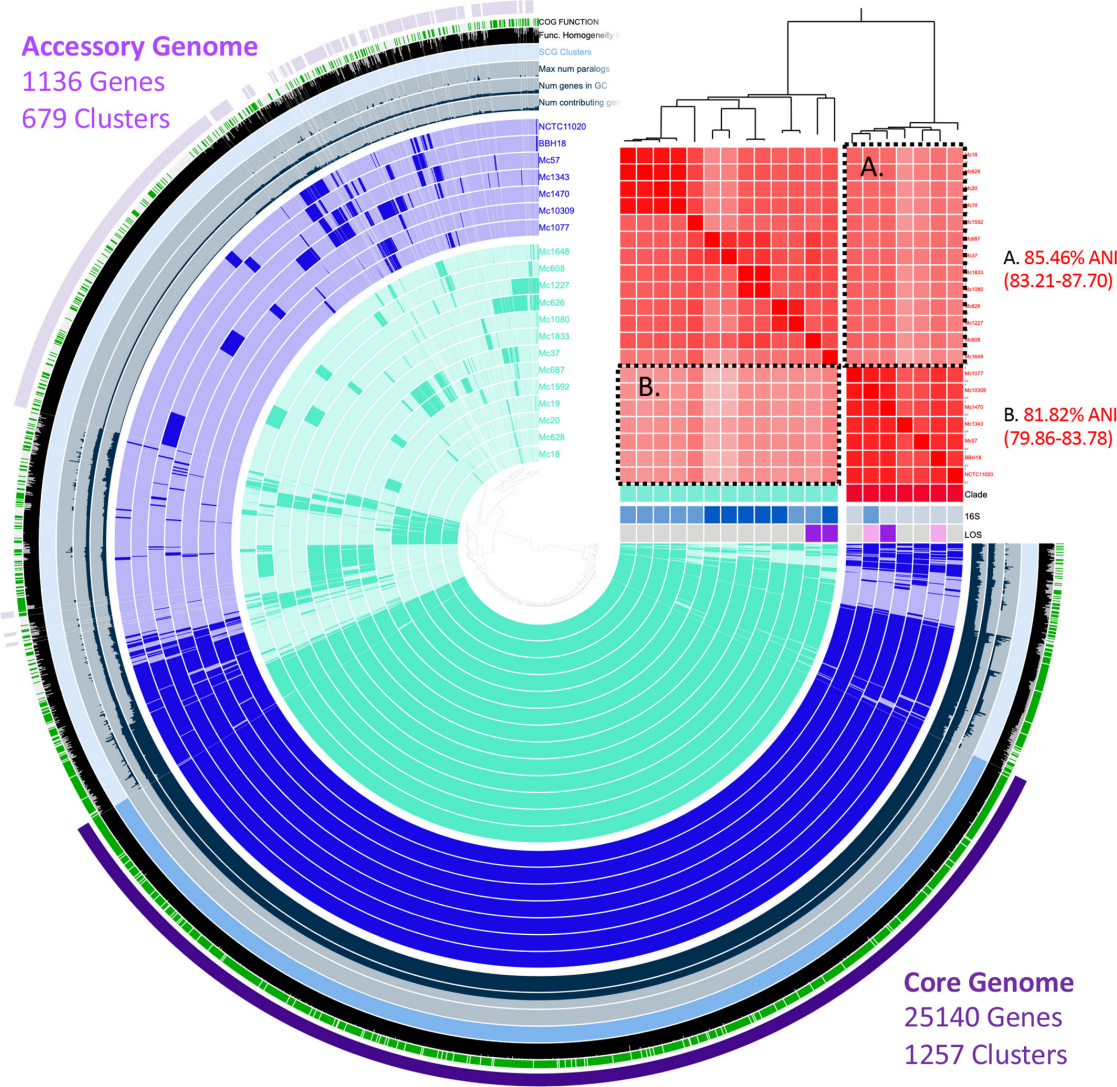
Pangenome of *M. catarrhalis *. Red-red clade, 93.91 % (91.49–96.32); blue to blue clade, 89.15 % (84.88–93.41); red to blue (box A in the figure); and blue to red (box B) are shown in the figure.

The *M. catarrhalis* pangenome is shown in Fig. 3. A total of 36 535 genes were used in the analysis, resulting in 2776 gene clusters. The core genome (genes occurring in all strains) was 68.8 %, comprising 25 140 genes in 1257 clusters. Genes that occurred in less <15 % of isolates (*n*=3) equated to 1136 genes in 679 clusters.

The functional enrichment analysis highlights the presence of multiple virulence genes that differentiate clade one and two (Table 4). There were 10 genes that were present in all seven clade one *M. catarrhalis* isolates, but were absent from all 13 clade two *M. catarrhalis* isolates. Genes only present in clade one seem to be involved in adaptation and the formation of resistance mechanisms. *rayt* encodes a rep element-mobilizing transposase that catalyses cleavage and recombination of bacterial interspersed mosaic elements (BIMEs), which may be important in the creation of BIME variability and amplification. BIMEs also act as binding sites for several proteins and are thought to be involved in organization of the chromosome and transcription termination [50]. Moreover, many of the genes in this clade are involved in the biosynthesis of antibiotics. For instance, the alkaline phosphatase (AlkP) family catalyse the hydrolysis of polyphosphates and phosphonates into antibacterial agents. Enzymes in this family are known to be involved in the biosynthesis of antibiotics such as streptomycin, bialaphos and mitomycin [51, 52]. Furthermore, PhoX family phosphatases catalyse the hydrolysis of phosphomonoester to a phosphate [53], whilst membrane-associated phospholipid phosphatases have a similar function [54]. As the name suggests, the ABC-type phosphate transport system and its ATPase component transports phosphate into the cell and hydrolyse it [55]. Restriction endonuclease Mrr cleaves DNA leaving double-stranded fragments with terminal 5'-phosphates. Mrr appears to be a final component of a bacterial SOS response to DNA damage. It is also an essential mechanism in generating genetic variability that facilitates adaptation and the development of resistance [56–58].

**Table 4.** Functional enrichment analysis Only significant data are reported in this table (those with a *P* value <0.05). All had *P* values of 0.00, corrected *P* values of 0.03 and an enrichment score of 3.49.

Clade	COGs function	Portion occurrence in group	Portion occurrence outside of group	Occurrence in group	Occurrence outside of group	Gene clusters IDs	Core in group	Core
C2	ABC-type bacteriocin/lantibiotic exporters, contain an N-terminal double-glycine peptidase domain	1	0.14	13	1	GC_00001559, GC_00002662	TRUE	FALSE
C2	Uncharacterized membrane protein YjiH, contains nucleoside recognition GATE domain	1	0	13	0	GC_00001582	TRUE	FALSE
C2	Predicted transposase YdaD	1	0	13	0	GC_00001531, GC_00001547	TRUE	FALSE
C2	DNA-binding transcriptional regulator, XRE-family HTH domain	1	0.14	13	1	GC_00001561, GC_00002218, GC_00002755	TRUE	FALSE
C2	Uncharacterized membrane protein YczE	1	0.14	13	1	GC_00001541	TRUE	FALSE
C2	Protein involved in initiation of plasmid replication	1	0.14	13	1	GC_00001513, GC_00001543, GC_00001550, GC_00001716, GC_00002180, GC_00002518, GC_00002635, GC_00002672	TRUE	FALSE
C2	Cys-tRNA(Pro) deacylase, prolyl-tRNA editing enzyme YbaK/EbsC	1	0	13	0	GC_00001567	TRUE	FALSE
C2	Phage-related protein, tail component	0.92	0.14	12	1	GC_00000083, GC_00001528, GC_00001843, GC_00002183, GC_00002230, GC_00002339	FALSE	FALSE
C2	Predicted epimerase YddE/YHI9, PhzF superfamily	1	0	13	0	GC_00001569	TRUE	FALSE
C2	Cell division protein DamX, binds to the septal ring, contains C-terminal SPOR domain	1	0	13	0	GC_00001573	TRUE	FALSE
C2	NADP-dependent 3-hydroxy acid dehydrogenase YdfG	1	0	13	0	GC_00001588	TRUE	FALSE
C2	DNA-binding transcriptional regulator, XRE family	1	0	13	0	GC_00001511	TRUE	FALSE
C2	Uncharacterized conserved protein, phosphatidylethanolamine-binding protein (PEBP) family	1	0	13	0	GC_00001584	TRUE	FALSE
C1	REP element-mobilizing transposase RayT	1	0	7	0	GC_00001768, GC_00002736	TRUE	FALSE
C1	ABC-type phosphate transport system, permease component	1	0	7	0	GC_00001724, GC_00001726	TRUE	FALSE
C1	Membrane-associated phospholipid phosphatase	1	0	7	0	GC_00001777	TRUE	FALSE
C1	Predicted pyrophosphatase or phosphodiesterase, AlkP superfamily	1	0	7	0	GC_00001732	TRUE	FALSE
C1	Secreted phosphatase, PhoX family	1	0	7	0	GC_00001783	TRUE	FALSE
C1	Predicted endonuclease, GIY-YIG superfamily	1	0	7	0	GC_00001627	TRUE	FALSE
C1	ABC-type spermidine/putrescine transport system, permease component II	1	0	7	0	GC_00001725	TRUE	FALSE
C1	Restriction endonuclease Mrr	1	0	7	0	GC_00001772	TRUE	FALSE
C1	ABC-type phosphate transport system, ATPase component	1	0	7	0	GC_00001727	TRUE	FALSE
C1	DNA modification methylase	1	0.08	7	1	GC_00001685	TRUE	FALSE
C1	Dienelactone hydrolase	1	0	7	0	GC_00001721	TRUE	FALSE

Colour highlights the genes present in all isolates in one clade but completely absent in the other clade. Two separate colours were used to differentiate those in clade one (red) and those in clade two.(blue).

There were eight genes that were present in all 13 clade two *M. catarrhalis *, but were absent in all 7 clade one *M. catarrhalis *. Genes only present in clade two are involved in stress responses and replication. For instance, *ydaD* is induced as a response to different stress conditions such as heat shock, oxidative stress, glucose limitation and oxygen limitation [59]. Furthermore, YdfG produces a dehydrogenase/reductase that catalyses oxidation–reduction reactions, this counteracts oxidative stress [60]. The DNA-binding transcriptional regulators, the XRE family, have been shown to play a role in oxidative and high temperature stress tolerance [61]. y*jiH* encodes a nucleoside recognition pore and gate family inner membrane transporter and YbaK functions *in trans* to edit the amino acid from incorrectly charged Cys-tRNA(Pro) via a Cys-tRNA(Pro) deacylase activity [62, 63]. YddE/YHI9 epimerases (part of the PhzF family) are involved in the production of phenazine derivative antibiotic compounds [64], whilst DamX protein plays a role in DNA adenine methylation, carbohydrate metabolism and tRNA charging [65].

### Antimicrobial-resistance (AMR) profiles

No AMR genes were detected using srst2 with the associated ARGannot. ARGannot uses an extensive list of antibiotic-resistance genes (*n*=1689) collected from published data and online resources with the nucleotide and protein sequences taken from the NCBI GenBank database including *bla* genes [66]. However, ARGannot is not specific for *M. catarrhalis* so may not include *bla BRO*, which comparison with other β-lactamases suggests is unique [67]. Again, whilst not specific for *M. catarrhalis *, ResFinder identifies the presence of an extensive list of acquired AMR genes and is continuously updated as new resistance genes are identified. β-Lactam-resistance genes were found in all 18 isolates (100%), with these genes having predicted phenotypes for resistance against ampicillin, penicillin, piperacillin and amoxicillin [68]. The production of β-lactamase is known as a leading source of resistance for *M. catarrhalis *. This enzyme digests β-lactam antibiotics rendering them ineffective, conferring resistance to antibiotics such as penicillins and cephamycins. *M. catarrhalis* produces two distinct β-lactamases: BRO-1 and BRO-2 [69]. A total of 72 % (13/18) of isolates were positive for the β-lactamase-encoding gene (*bla) BRO-1* and 28 % (5/18) for *bla BRO-2*. No isolates had both BRO-1 and BRO-2 genes.

Additionally, srst2 was used to map the *bla* gene in all isolates again to confirm gene presence and BRO-1 or BRO-2 status. Gene mapping supported the findings from ResFinder, showing all 18 isolates were *bla* positive. The 100 % prevalence of β-lactamase resistance genes is comparable to the 98 % seen in other publications [49], and in agreement with the widespread nature of β-lactam resistance in *M. catarrhalis *. We observed a lower proportion of *bla BRO*-1 isolates than previously reported: 72 % (13/18) in this dataset versus the 91 % previously seen [49]. It remains to be seen whether this difference in BRO-1 and BRO-2 prevalence is a reflection of differences between carriage and disease. However, it potentially enforces the importance of β-lactamase resistance as a virulence factor giving *M. catarrhalis* a potential clinical edge for causing disease; particularly as *bla BRO*-1 is the stronger of the two, producing more β-lactamase than BRO-2 counterparts [67, 70, 71]. What is interesting is that prior data has suggested that BRO-2 isolates are associated with the 16S type 1 lineage [28]; however, this was not seen here (Table 3). To confirm the expression of β-lactamase, isolates were tested with β-lactamase identification sticks. All 18 isolates tested positive for the production of β-lactamase. Fortunately, β-lactamase production does not automatically mean resistance, β-lactamase does not render all β-lactam antibiotics ineffective; second-, third- and fourth-generation cephalosporins are still effective [72].

When tested phenotypically, all isolates were sensitive to amoxicillin/clavulanic acid, cefotaxime, ceftriaxone, erythromycin, tetracycline, chloramphenicol, ciprofloxacin and meropenem. These antibiotics were used to test resistance to cephalosporins, macrolide, tetracycline, chloramphenicol, fluoroquinolones and carbapenem antibiotics, whilst amoxicillin/clavulanic acid is a combination antibiotic containing potassium clavulanate, a β-lactamase inhibitor; thus, is commonly used to treat β-lactam-resistant bacteria. Although cefotaxime and ceftriaxone are β-lactam antibiotics, they are third-generation cephalosporins, which are known to still be effective for *M. catarrhalis *, so data are in keeping with current literature.

### Distribution of virulence factors

Table 3 highlights the presence and absence of virulence genes tested in all isolates, including reference strains BBH18 and NCTC 11020. *copB*, *ompCD*, *ompE*, *ompG1a*, *ompG1b*, *mcaP*, *m35*, *tbpA*, *lbpA*, *tbpB*, *lbpB*, *msp75*, *msp78*, *afeA*, *pilA*, *pilQ*, *pilT*, *oppA* and *sbp2* were present in 100 % of the carriage isolates, which is comparable to other studies, most of which focus on clinical isolates [21, 22, 28, 73, 74]. In total, 88.9 % (16/18) of isolates contained *mid/hag*, this is in agreement with prior publications that report *mid/hag* to be present in 80 % of child carriage isolates [27] and in up to 100 % of clinical strains [29]. The two isolates ‘missing’ did have signs of the gene; however, they were below the coverage cut-off. This either means the gene is only partially present or could mean that the gene is present but is altered. Less is published on the prevalence of *mcmA* and *mclS*; however, these were also present in all isolates here.

Due to the overlap of gene sequence between *uspA1* and *uspA2H*, and *uspA2* and *uspA2H*, it was not possible to confirm the presence or absence of *uspA* genes using read mapping (Table 5). To try to clarify gene presence, coverage was visually inspected in Tablet v. 1.19.09.93 [75] and *in silico* PCR using published primers was attempted; however, results remain unclear.

**Table 5.** *uspA* read mapping results (percentage of gene coverage)

Accession numbers	Gene	Percentage of gene coverage																	
		57	1343	1470	10 309	1077	1648	608	1227	626	1080	1833	37	687	1592	19	20	628	18
EU430059.1	*uspA1*	42.9	46.6	72.0	17.6	70.5	49.2	69.9	0.0	0.0	63.5	10.6	26.8	15.4	29.5	41.9	25.1	22.6	48.9
U61725.1		61.8	43.6	93.5	62.8	81.6	59.0	48.6	10.9	14.6	56.2	52.4	63.7	65.8	54.4	58.9	63.5	62.0	61.3
AF113610.1		96.4	79.0	73.3	76.7	69.3	42.8	53.0	9.5	7.6	52.2	48.8	49.1	54.7	42.3	44.9	45.4	45.9	41.1
AF352398.1		55.2	66.7	78.0	41.7	82.8	50.0	46.2	8.0	10.3	55.9	33.4	51.7	62.0	54.2	48.1	51.1	54.1	50.8
AY730666.1	*uspA2*	62.6	67.1	58.1	48.2	79.9	39.7	24.1	31.2	47.5	39.7	43.0	36.0	59.9	35.6	31.0	30.4	35.2	35.2
AF410950.1		24.9	43.2	30.8	43.5	53.6	34.4	57.9	38.1	31.4	25.9	31.8	39.6	35.6	33.4	32.4	33.1	34.8	32.5
AF352399.1		32.5	33.8	38.7	15.0	39.5	41.2	43.1	34.0	33.5	37.5	30.8	42.3	40.4	34.6	25.9	28.0	38.9	38.7
AF181073.1		99.0	47.9	29.3	36.6	98.4	50.3	37.9	30.0	33.3	33.8	41.9	36.2	33.6	47.0	28.8	28.3	32.8	31.2
AF113609.1		40.7	86.5	34.0	46.9	55.6	49.1	52.2	38.2	39.9	37.8	34.7	49.4	40.7	41.9	35.6	36.9	42.6	40.0
AF181075.1	*uspA2H*	88.9	75.1	100.0	100.0	83.7	65.0	73.1	24.6	23.7	65.5	66.3	63.1	63.0	80.8	64.6	68.5	75.0	73.4
DQ811779.1		63.8	55.4	71.3	73.8	77.9	54.9	58.4	19.1	26.9	77.5	73.2	63.4	24.9	31.4	33.3	38.6	47.6	31.7
AF410951.1		32.1	38.7	54.2	73.4	36.8	35.0	27.1	10.4	18.0	23.2	19.6	21.9	24.4	25.4	22.3	25.8	23.5	24.7
AF181074.1		29.4	44.9	78.6	54.4	39.1	31.9	23.9	13.7	13.9	20.4	26.7	22.8	24.3	36.6	29.0	31.9	29.6	33.5
AF181075.1		51.4	45.5	68.7	75.8	47.1	32.1	30.0	22.6	21.4	25.8	26.1	27.5	24.1	44.6	37.9	33.6	41.1	40.8

The *M. catarrhalis* two-partner secretion (TPS) system comprises MhaC (transporter), MhaB1 (exoprotein) and MhaB2 (exoprotein) [28, 76, 77]. Here, *mhaB1* was present in 44.4 % (8/18) of isolates, *mhaB2* was present in none of the isolates and *mhaC* was present in 55.6 % (10/18) of isolates. Other studies have shown both MhaB1 and MhaB2 expression in *M. catarrhalis* strain O35E; it is, therefore, interesting that *mhaB2* was not found in any of our isolates [76]. Our data further contrast previous research, which showed 100 % presence of *mhaC*, *mhaB1* and *mhaB2* in isolates, although these were of clinical origin [28, 77]. Overall, *mhaB1* and *mhaC* were present in 30 % (3/10) and 50 % (5/10) of isolates from children and 62.5 % (5/8) and 62.5 % (5/8) of isolates from adults, respectively. When distribution is considered by age range, *mhaB1* and *mhaC* were present in 33.3 % (2/6) and 50 % (3/6) of isolates from 0 to 4 year olds, 25 % (1/4) and 50 % (2/4) of isolates from 5 to 16 year olds, 75 % (3/4) and 75 % (3/4) of isolates from 17 to 59 year olds, and 50 % (2/4) and 50 % (2/4) of isolates from 60+ year.

Both *tbpA* and *tbpB* were present in all isolates, which is comparable to other studies [28, 78]. Again, in agreement with prior research, *lbpA* and *lbpB* were present in 100 % of isolates [28, 79]. *msp22* was present in 94.4 % (17/18) of isolates, which is lower than the 100 % found in other studies [80]; however, the isolate classed as negative for *msp22* did have a coverage of 82 %, which was below the cut-off. When visually inspected in Tablet v. 1.19.09.93 [75] in the isolates ‘missing’ *msp22*, the gene appeared partially present; however, it showed a lack of coverage in the final ~80 bp. Overall, 90 % (9/10) of isolates from children and 100 % (8/8) of isolates from adults possessed *msp22*. When distribution is considered by age range 83.3 % (5/6) of isolates from 0 to 4 year olds, 100 % (4/4) of isolates from 5 to 16, 100 % (4/4) of isolates from 17 to 59 and 100 % (4/4) of isolates from 60+ year olds possessed *msp22*.

*modM* was present in 61.1 % (11/18) of isolates, much lower than previous reports suggesting *modM* to be present in 100 % of the carriage and clinical strains tested [81]. Overall, 70 % (7/10) of isolates from children and 62.5 % (5/8) of isolates from adults possessed *modM* here. When distribution is considered by age range, 50 % (3/6) of isolates from 0 to 4 year olds, 75 % (3/4) of isolates from 5 to 16, 25 % (1/4) of isolates from 17 to 59 and 100 % (4/4) of isolates from 60+ year olds possessed *modM*. It is unclear why the data from this study should be different to prior publication [81].

From the data presented here, there appears to be no association between LOS type, MLST and 16S type and the presence or absence of any of the virulence factors; however, the limited number of isolates analysed impedes the conclusions that can be drawn. Further research is needed with a greater number of samples. An important caveat that should be made is that just because homology exists between a reference gene and an isolate’s genome, it does not mean that the gene exists in an ORF in that genome. The results here highlight potential gene presence but not evidence of intact ORFs.

### Conclusion

This is the only study, to our knowledge, to focus on the epidemiology of and distribution of virulence factors in *M. catarrhalis* carriage isolates from all ages. As carriage is considered a prerequisite for disease [31], it is important to better understand the epidemiology of *M. catarrhalis* in the community. Especially as no particular type, subpopulation nor strain has been implicitly associated with disease or virulence. The key limitation of this study is the low number of isolates analysed, which restricts how accurately the data reflects the epidemiology and distribution of virulence factors for *M. catarrhalis *. Therefore, this paper should be followed by further analysis on a larger set of community isolates. This study does, however, provide a novel and timely insight into the types of *M. catarrhalis* carried, the AMR profiles of such isolates and the distribution of virulence factors, which can be used to aid our understanding of the disease potential of community isolates and to inform vaccine development. Further to this, a comparison of carriage and clinical isolates would be beneficial and likely to provide additional data to facilitate a better understanding of the differences between carriage and disease and the identification of markers of pathogenic strains of *M. catarrhalis *.
